# Functions of Epimedin C in a zebrafish model of glucocorticoid‐induced osteoporosis

**DOI:** 10.1111/jcmm.18569

**Published:** 2024-07-28

**Authors:** Xiaoyang Zhou, Kai Lian, Junjie Jia, Xue Zhao, Peng Duan, Jiaolong Huang, Yihua Shi

**Affiliations:** ^1^ Department of Orthopedics, Xiangyang No. 1 People's Hospital Hubei University of Medicine Xiangyang China; ^2^ Key Laboratory of Zebrafish Modeling and Drug Screening for Human Diseases of Xiangyang City, Department of Obstetrics and Gynaecology, Xiangyang No. 1 People's Hospital Hubei University of Medicine Xiangyang China

**Keywords:** bone mineralization, Epimedin C, glucocorticoid‐induced osteoporosis, osteoprotective effect, traditional Chinese medicine, zebrafish model

## Abstract

Epimedium is thought to enhance the integrity of tendons and bones, ease joint discomfort and rigidity and enhance kidney function. Although glucocorticoids are commonly used in clinical practice, the mechanism by which the active compound Epimedin C (EC) alleviates glucocorticoid‐induced osteoporosis (GIOP) is not well understood. The therapeutic potential of EC in treating GIOP was evaluated using alizarin red S staining, calcein immersion and fluorescence imaging, and bone mineralization, bone mass accumulation and bone density in zebrafish larvae were determined. Using the Kyoto Encyclopedia of Genes and Genomes (KEGG) analysis, the key signalling pathways related to bone development were identified. A protein–protein interaction network (PPIN) was constructed to identify osteoclast characteristic genes and the findings were verified using real‐time quantitative PCR (RT‐qPCR). The bone tissue damage caused by prednisolone was reduced by EC. It also altered physiological processes, improved bone density, boosted mineralization and increased bone mass and activity. Subsequent empirical investigations showed that EC impacted the major signalling pathways involved in bone development, such as osteoclast differentiation, oestrogen, MAPK, insulin resistance, PPAR and AMPK signalling pathways. It also decreased the expression of genes typical of osteoclasts. The results of our study uncover a previously unknown function of EC in controlling bone formation and emphasize the potential of EC as a therapeutic target. The osteoprotective effect of EC indicates its potential as a cost‐effective strategy for treating GIOP.

## 1 BACKGROUND

Osteoporosis (OP) is a common chronic bone disease affecting an increasing number of people worldwide. It is characterized by reductions in bone strength and bone density, together with bone tissue deterioration, thus increasing the likelihood of bone fractures.^1^ Glucocorticoids (GCs), commonly prescribed for conditions such as rheumatoid arthritis, asthma and allergic rhinitis, have detrimental effects on bone health by reducing bone formation and increasing bone resorption.^2^, ^3^ GC‐induced OP (GIOP) is a condition that occurs as a result of the treatment, and it is defined by a reduction in both bone mass and structural strength. Individuals who are affected may suffer from lumbar pain and have an increased susceptibility to fractures and kyphosis, all of which can significantly compromise their physical and psychological well‐being. Given the growing number of elderly individuals, it is crucial to identify efficacious interventions for the prevention and treatment of GIOP.

Bisphosphonates, including etidronate disodium (ED), are synthetic compounds that attach to the bone matrix, offering a more targeted strategy when treating disorders of the bone.^4^ They act by preventing bone resorption and promoting bone formation, thereby increasing bone density, relieving bone pain and reducing the likelihood of fractures in affected individuals.^5^ Upon absorption by osteoclasts in the bone matrix, bisphosphonates can induce apoptosis in these cells, thereby disrupting their communication with the bone matrix and decreasing bone resorption.^6^ As its mechanism and efficacy in treating OP are relatively well understood, ED is frequently employed as a positive control in scientific investigations. Additionally, ED has the potential to disrupt the development, movement and maturation of precursors for osteoclasts.^7^ Numerous studies have confirmed the significant reduction in bone resorption resulting from bisphosphonate use.^8^ Nevertheless, whereas bisphosphonate use leads to an evident augmentation in bone mass, there is still a discernible association between bisphosphonates and elevated susceptibility to specific types of fractures.^9^ Owing to the limitations associated with the current treatments for OP, there has been a growing interest in natural product‐based therapies. High‐throughput screening of natural products can serve as a crucial strategy to identify active compounds to be developed as anti‐OP drugs.

Epimedium, a traditional Chinese herbal medicine, is known for its diverse healing properties. It has the effects of strengthening tendons and bones, alleviating wind and dampness symptoms and improving kidney function.^10^, ^11^ The earliest references to Epimedium appear in ‘The Classic of Herbal Medicine’, and its applications have been documented in various pharmacopoeial texts over time.^12^ Multiple studies have shown that Epimedium can stimulate the growth of new bone and prevent bone loss. This helps maintain a balance where the formation of new bone exceeds the rate of bone degradation.^13^, ^14^ This property is particularly advantageous in the prevention and treatment of OP. Recent studies have demonstrated the potential of Epimedin C (EC), an active ingredient of Epimedium, in treating OP, especially GIOP.^15^ Nevertheless, the exact biochemical process by which EC preserves bone remains poorly understood. Zebrafish are commonly utilized in bone research due to their various advantages, including their tiny size and clear embryos. Additionally, zebrafish have similar ossification processes to mammals, which allows for the convenient observation of osteoporosis phenotypes induced by glucocorticoids. In this study, we assessed the efficacy of EC using zebrafish larvae exposed to prednisolone (PN). Transcriptomic sequencing demonstrated that EC could ameliorate the osteoporotic phenotype via downregulation of osteoclast characteristic genes.

## 2 METHODS

### 2.1 Chemicals and reagents

Epimedin C (EC) was purchased from Bidepharm (Shanghai, China), while glycerol was from Solarbio (Beijing, China), potassium hydroxide (KOH) from Sigma (St. Louis, MO, USA), hydrogen peroxide (H_2_O_2_) from Tianli (Tianjin, China), prednisolone from Adamas‐beta (Shanghai, China), ED from Aladdin (Shanghai, China), alizarin red S staining solution from Beyotime (Shanghai, China), calcein from Sigma and 4% paraformaldehyde from Beyotime.

### 2.2 Experiments with zebrafish

AB wild‐type zebrafish (*Danio rerio*) were maintained in a flow‐through aquarium system in our laboratory for a 14 h light/10 h dark daily cycle at 28 ± 0.5°C. Live brine shrimp (*Artemia nauplii*) were provided twice daily to the zebrafish to meet their dietary requirements.

Before initiating experiments, both male and female zebrafish, aged 3–4 months, were reared in separate glass tanks for 10 days for acclimatization to the laboratory setting.^16^ Mature fish underwent biweekly breeding sessions to prepare them for reproduction before the exposure study. For subsequent experiments, the zebrafish were placed in a specialized breeding tank in a female: male ratio of 1:2.^17^ Individual compartments with dividers were used to keep the fish isolated overnight. These barriers were removed at the break of the day to promote natural mating activity. Eggs that were laid and fertilized post‐mating were collected quickly, typically within 30 minutes of spawning.^18^, ^19^

The study examined neural activity and physiological changes in zebrafish embryos. Various factors, including movement behaviour, mortality rate, heart rate, blood flow and blood flow velocity, were measured at 9 days after fertilization (dpf). The experimental protocol and procedures received approval from the Biological Basic Research Ethics Committee of our university (Review Approval Number: XYYYE20230055).

### 2.3 Grouping and drug intervention

Zebrafish larvae at 4 dpf were raised in six‐well plates with each well containing 20 larvae. Medium (8 mL per well) containing different drugs was added to the wells. During this period, the larvae did not require feeding. Zebrafish larvae in the PN groups were exposed to different concentrations of PN ranging from 5, 25, 45, 65 and 85 μmol/L, whereas those in the control group were grown in a medium containing 0.5% dimethyl sulfoxide (DMSO). A supplementary group of larvae was subjected to positive drug treatment by administering a combination of 15 μg/mL ED and 25 μmol/L PN. The larvae in the EC groups received different amounts of EC (1, 5, and 10 μmol/L), along with 25 μmol/L PN. The media in the wells were renewed daily by replacing half of the volume. At 9 dpf, the zebrafish larvae were rendered unconscious using MS222, and then humanely killed and collected for analysis.

### 2.4 Alizarin red S staining

Zebrafish larvae (9 dpf) were fixed in a 4% paraformaldehyde solution overnight. The following day, after the removal of the surplus fluid, a bleaching solution comprising 3% H_2_O_2_ and 2% KOH was added to lighten the melanin in the heads and bodies of the larvae. The mixture was permitted to react for 8 hours. The degree of bleaching was monitored with a stereomicroscope (Olympus, Japan) until the larvae became translucent. At this point, the surplus liquid was poured off and the specimens were immersed in a 0.005% solution of alizarin red S for 12 hours for staining. To remove excess staining solution, a sequential washing process was performed using a solution consisting of a mixture of 1% KOH and glycerol in different ratios of 3:1, 1:1 and 1:3. The zebrafish larvae were examined after washing. The stained skulls were examined and imaged using a stereomicroscope. The images were analysed using the imaging software Image‐Pro Plus 6.0.

### 2.5 Calcein immersion and fluorescence imaging

The amount of calcified bone in the skeletal structure was measured using calcein, a fluorescent molecule with a specific affinity for calcium. This indicator is closely related to bone density.^20^ After 5 days of treatment, larvae from each group were randomly chosen for further analysis. The process involved immersing the specimens in 0.2% calcein, as previously described.^21^ Fluorescence imaging was performed using a fluorescence microscope (Guangzhou, China) equipped with the MShot Image Analysis System (Guangzhou, China). The zebrafish vertebrae V1‐V3 were selected for observation and quantitative analysis of the fluorescence intensity.

### 2.6 Locomotory behaviour in zebrafish larvae

At 9 dpf, individual zebrafish larvae were placed in separate wells of a 24‐well plate, ensuring 1 larva per well. For additional research, only larvae with normal morphology were selected. The selected specimens were placed in the Zebrafish Tracking System observation room (ViewPoint, France) and allowed at least 5 minutes to acclimate. The larvae were thought to be prepared for analysing their swimming patterns after they had acclimated. ViewPoint Application Manager software (ViewPoint, France) was used to document the swimming activity of each group of larvae for a continuous period of 30 min. The documentation included alternating exposure to light and dark phases that lasted 5 min each.

### 2.7 RNA sequencing (RNA‐Seq) analysis

Samples of 9 dpf zebrafish larvae were collected and placed in centrifuge tubes and centrifuged to separate the supernatant as much as possible. The samples were transported on dry ice to Wuhan Huada Gene Technology Service Co., Ltd. for high‐throughput screening and RNA extraction and purification. Each experimental condition was replicated in four independent samples to ensure the robustness and reliability of the data collected. The raw data from the RNA‐Seq analysis have been deposited in the NCBI Sequence Read Archive (SRA) and are accessible under the BioProject accession number PRJNA1100624, ensuring data transparency and reproducibility.

RNA extraction and purification were performed prior to sequencing. The analysis of differentially expressed genes (DEGs) was performed on the Dr. Tom Multi‐omics Data mining system (https://biosys.bgi.com). DEGs were identified based on the criteria of the absolute value of log2‐ratio ≥0 and a *P*‐value ≤0.05. The biological significance of DEGs was analysed using the Gene Ontology (GO) and Kyoto Encyclopedia of Genes and Genomes (KEGG) databases to determine the associated biological processes. *p* < 0.05 was used to determine the significance of GO and KEGG pathway enrichment.

To explore the interactions among the proteins encoded by DEGs, a protein–protein interaction network (PPIN) was constructed using the STRING database with visualization in Cytoscape.

### 2.8 Gene expression analysis

To determine the transcriptional expression of representative genes, RNA was extracted from 30 zebrafish larvae (CWBIO, Jiangsu, China) that were randomly selected at 9 days of maturity and placed in each group. The RNA was extracted using TRIzol reagent and the purity and concentration of the RNA were assessed by absorbance measurements, with A260/A280 values between 1.8 and 2.2 deemed to be of acceptable purity. The RNA was reverse‐transcribed to cDNA using a HiScript III RT SuperMix for qPCR (+gDNA wiper) (Vazyme Biotech, Nanjing, China).

Real‐time quantitative PCR (RT‐qPCR) was performed using Taq Pro Universal SYBR qPCR Master Mix (Vazyme Biotech), and all primers were designed by Sangon Biotech Co., Ltd. (Shanghai, China). Three biological replicates and two technical duplicate wells were used for all experiments. Differences were calculated using the 2^−ΔΔCT^ comparative quantitation method and β‐actin was used as an internal control.

### 2.9 Statistical analysis

Data were statistically analysed and visualized using GraphPad Prism 8.0.2. Quantitative data are presented as mean ± standard deviation. One‐way analysis of variance (ANOVA) followed by the post hoc least significant difference (LSD) multiple comparison test was used to determine statistical significance. A *P*‐value <0.05 was considered statistically significant.

## 3 RESULTS

### 3.1 Determination of the optimal PN concentration for modelling

**FIGURE 1:**
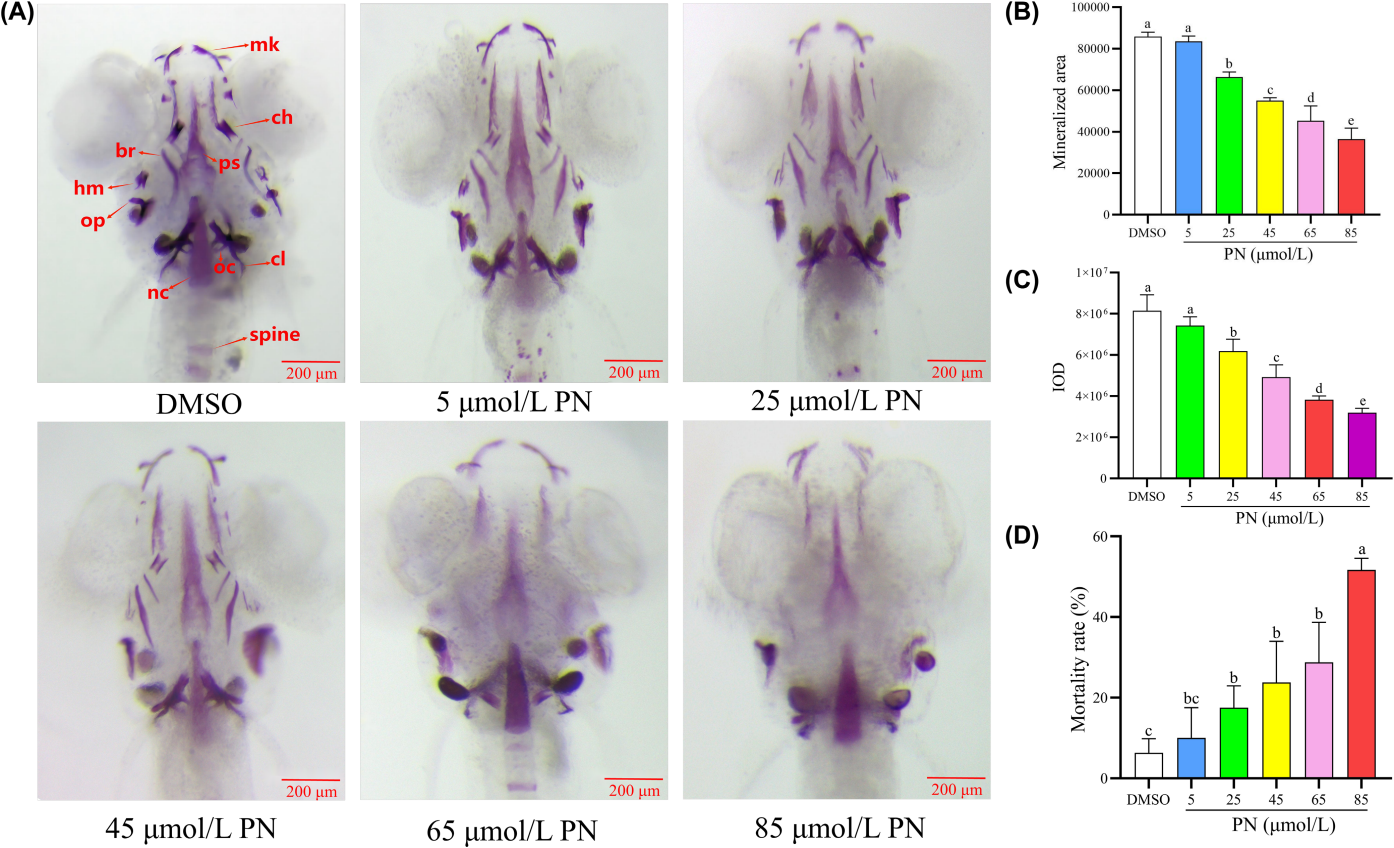
Effect of different concentrations of PN on bone mineralization and bone mass accumulation in the skulls of zebrafish larvae (*n* = 6). (A) Zebrafish larvae at 9 dpf were treated with different PN concentrations and were stained with alizarin red S. Images show the ventral view of zebrafish skulls. (B) Mineralized area assessed by measuring the areas of the stained skulls. (C) IOD of stained skulls measured using quantitative analysis. (D) Mortality rate of zebrafish larvae increases with increasing PN concentration. br, branchial ray; ch, ceratohyal; cl, cleithrum; hm, hyoid mandibular arch; mk, Meckel's cartilage; nc, notochord; oc, occipital bone; op, operculum; ps, parasphenoid bone. Scale bar: 200 μm. Different letters above the error bars indicate significant differences (*p* < 0.05) among the groups.

Zebrafish larvae were exposed to varying concentrations of PN to establish a dependable model of GIOP. The average staining area and integrated optical density (IOD) of the larval skulls were measured after the exposure. As seen in Figure 1, larvae in the 25, 45, 65 and 85 μmol/L PN groups showed a notable reduction in the average staining area and IOD compared with those in the control group, and the degree of reduction was concentration dependent (*p* < 0.05).

Furthermore, the death rate of the larvae increased in correspondence with the PN concentration, indicating a dependence on concentration. To ensure the efficacy of PN treatment and lower the mortality rate in zebrafish larvae, the ideal concentration for constructing a reliable GIOP model was found to be 25 μmol/L.

### 3.2 Effect of EC on bone mineralization and bone mass accumulation in the skulls of zebrafish larvae

**FIGURE 2:**
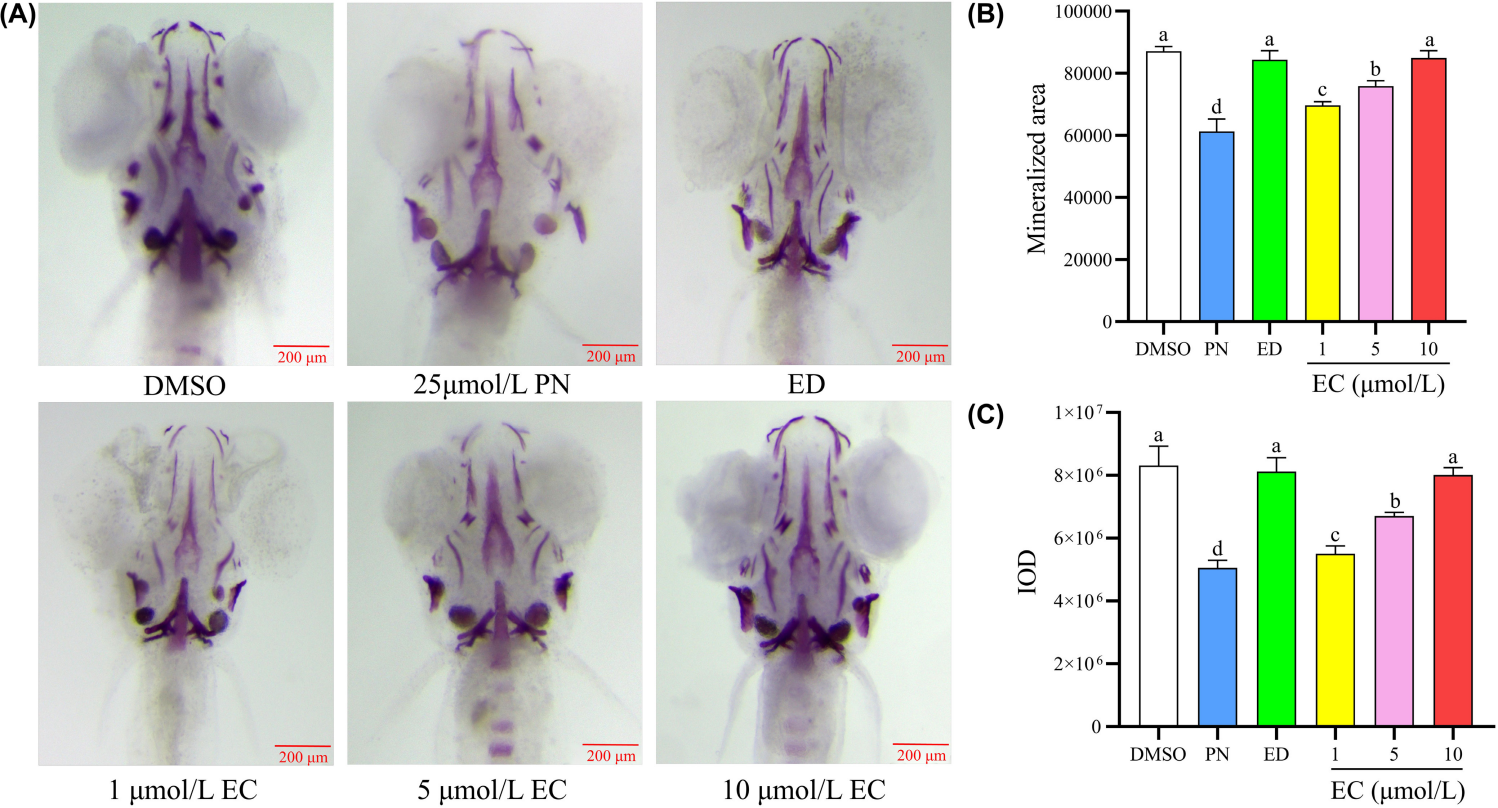
Effect of different concentrations of EC on bone mineralization and bone mass accumulation in the skulls of zebrafish larvae (*n* = 6). (A) Zebrafish larvae at 9 dpf were treated with different EC concentrations and were stained with alizarin red S. Images show the ventral view of zebrafish skulls. (B) Mineralized area assessed by measuring the areas of the stained skulls. (C) IOD of the stained skulls measured using quantitative analysis. Scale bar: 200 μm. Different letters above the error bars indicate significant differences (*p* < 0.05) among the groups.

Figure 2 illustrates the effect of EC on the skulls of the zebrafish larvae. Compared with the control group, the 25 μmol/L PN group exhibited a significant reduction in the average staining area and IOD (*p* < 0.05). In comparison, the average staining area and IOD of the 15 μg/mL ED group were considerably higher than those of the 25 μmol/L PN group (*p* < 0.05). When EC was added at 1, 5 and 10 μmol/L, the average staining area and IOD were substantially greater than in the 25 μmol/L PN group (*p* < 0.05).

### 3.3 Effect of EC on the bone density of zebrafish larvae

**FIGURE 3:**
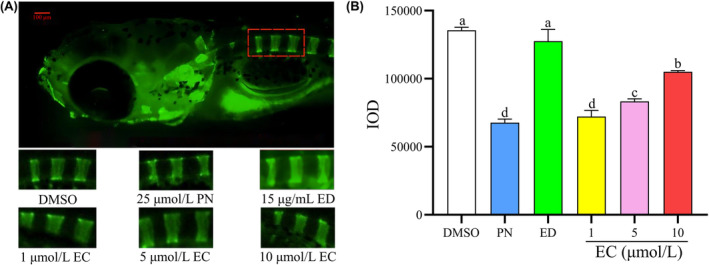
Effect of different concentrations of EC on IOD in zebrafish with GIOP. A zebrafish model of GIOP was established using 25 μmol/L PN. The six exposure groups included a control group, 25 μmol/L PN group, 15 μg/mL ED group and three EC‐treatment groups administered the corresponding treatment for 5 days (*n* = 6). (A) Fluorescence imaging after calcein immersion using fluorescence microscopy. (B) IOD of the one to three vertebral bones of zebrafish in different groups determined using quantitative analysis. Scale bar: 100 μm. Different letters above the error bars indicate significant differences (*p* < 0.05) among the groups.

Compared with that in the control group, treatment with 25 μmol/L PN significantly reduced vertebral fluorescence intensity, indicating bone density loss **(**Figure 3).

A concentration of 15 μg/mL of ED reversed the decrease in the intensity of fluorescence in the vertebrae. When compared to a concentration of 25 μmol/L of PN, a concentration of 1 μmol/L of EC did not cause a significant change in the intensity of fluorescence in the vertebrae of zebrafish larvae (*p* > 0.05). However, concentrations of 5 and 10 μmol/L of EC did reduce the decrease in vertebral bone density caused by PN (*p* < 0.05).These findings suggest that 5 and 10 μmol/L EC can protect against the detrimental effects of PN on bone density.

### 3.4 Effect of EC on the physiological functions of zebrafish larvae

**FIGURE 4:**
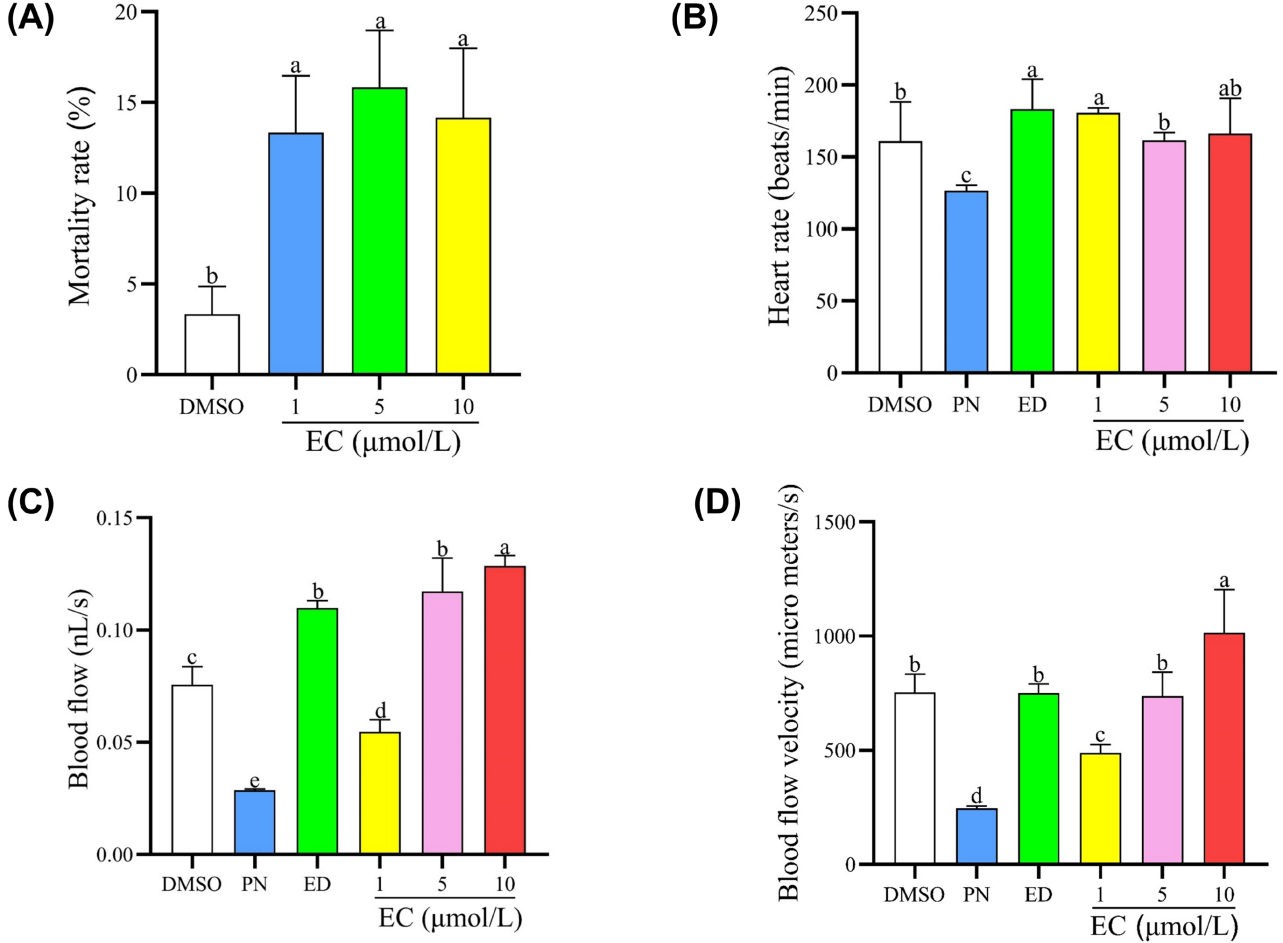
Physiological effects of different EC concentrations in zebrafish larvae. (A) The mortality rate of zebrafish larvae in the three EC‐treatment groups (*n* = 3). (B) The heart rate of zebrafish larvae in the different exposure groups (*n* = 8). (C) The blood flow of zebrafish larvae in the different exposure groups (*n* = 10). (D) The blood flow velocity of zebrafish larvae in the different exposure groups (*n* = 10). Different letters above the error bars indicate significant differences (*p* <0.05) among the groups.

The mortality rate of three EC‐treatment groups increased compared with the control group (*p* < 0.05); however, this increase was not concentration‐dependent (Figure 4A). Moreover, the heart rate, blood flow and velocity of blood flow decreased significantly in the 25 μmol/L PN group relative to the control group. However, these adverse effects could be reversed by treatment with 1, 5 and 10 μmol/L EC (*p* < 0.05) (Figure 4B–D).

### 3.5 Effect of EC on the locomotion of zebrafish larvae

**FIGURE 5:**
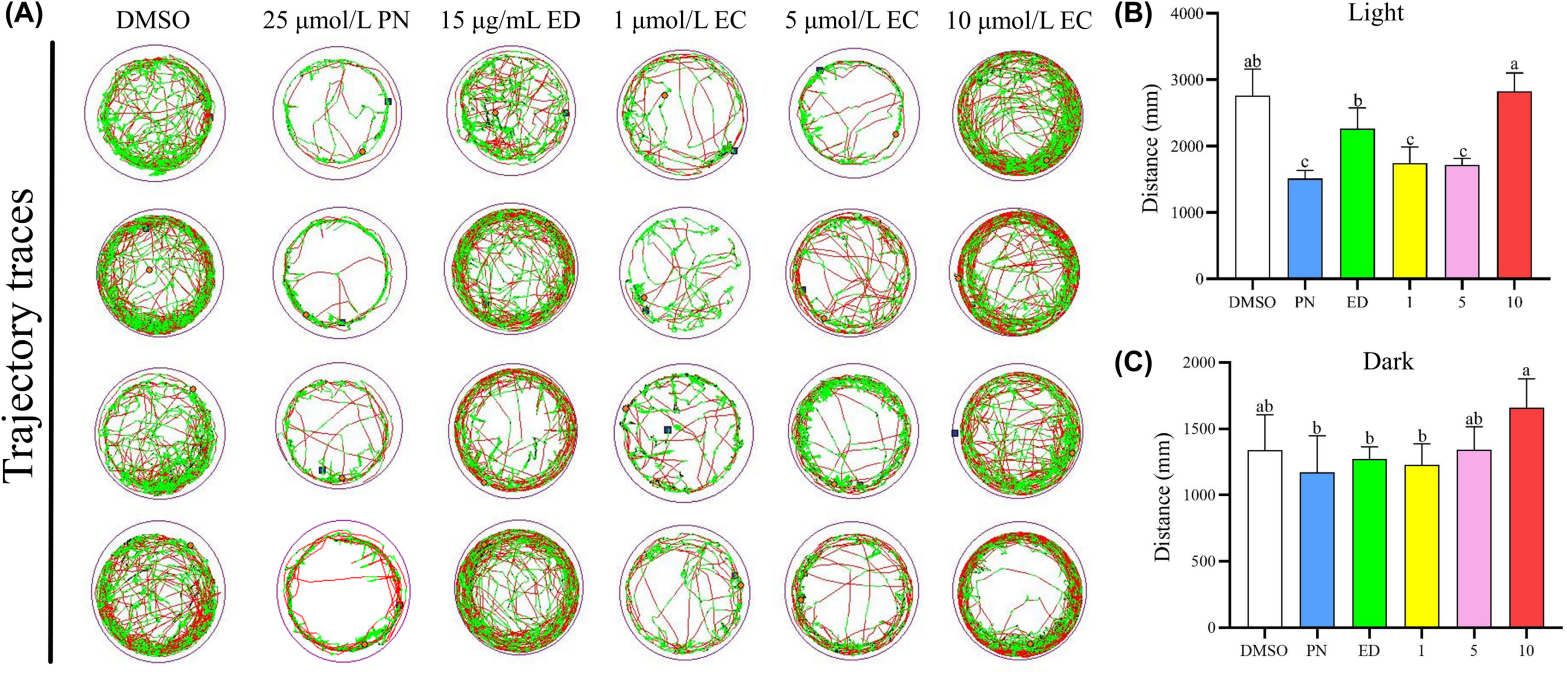
Neurobehavioral changes in zebrafish larvae induced by different EC concentrations (*n* = 4). (A) Representative trajectory traces of 9 dpf zebrafish larvae in six exposure groups for a continuous period of 30 minutes. (B) Total distances moved by 9 dpf zebrafish larvae during the light cycle. (C) Total distances moved by 9 dpf zebrafish larvae during the dark cycle. Different letters above the error bars indicate significant differences (*p* < 0.05) among the groups.

The total distances moved by the zebrafish larvae exposed to 25 μmol/L PN were significantly shorter than those of the larvae in the control group during the light cycle, as illustrated in Figure 5. During the dark cycle, while the movement distance of the PN‐treated group was reduced compared to the DMSO‐treated control group, this reduction did not reach statistical significance (*p* > 0.05). This suggests that the behavioural impact of PN may vary depending on the light conditions of the environment.

Furthermore, it was observed that the total movement distance of group administered 10 μmol/L EC was considerably greater in comparison to the group treated with 25 μmol/L PN during both the light and dark cycles (*p* < 0.05). The results of this study indicated that increasing the concentration of EC in zebrafish larvae might improve their locomotory capacity.

## 4 WHOLE TRANSCRIPTOME SEQUENCING DATA ANALYSIS

### 4.1 Transcriptome assembly and analysis

**FIGURE 6:**
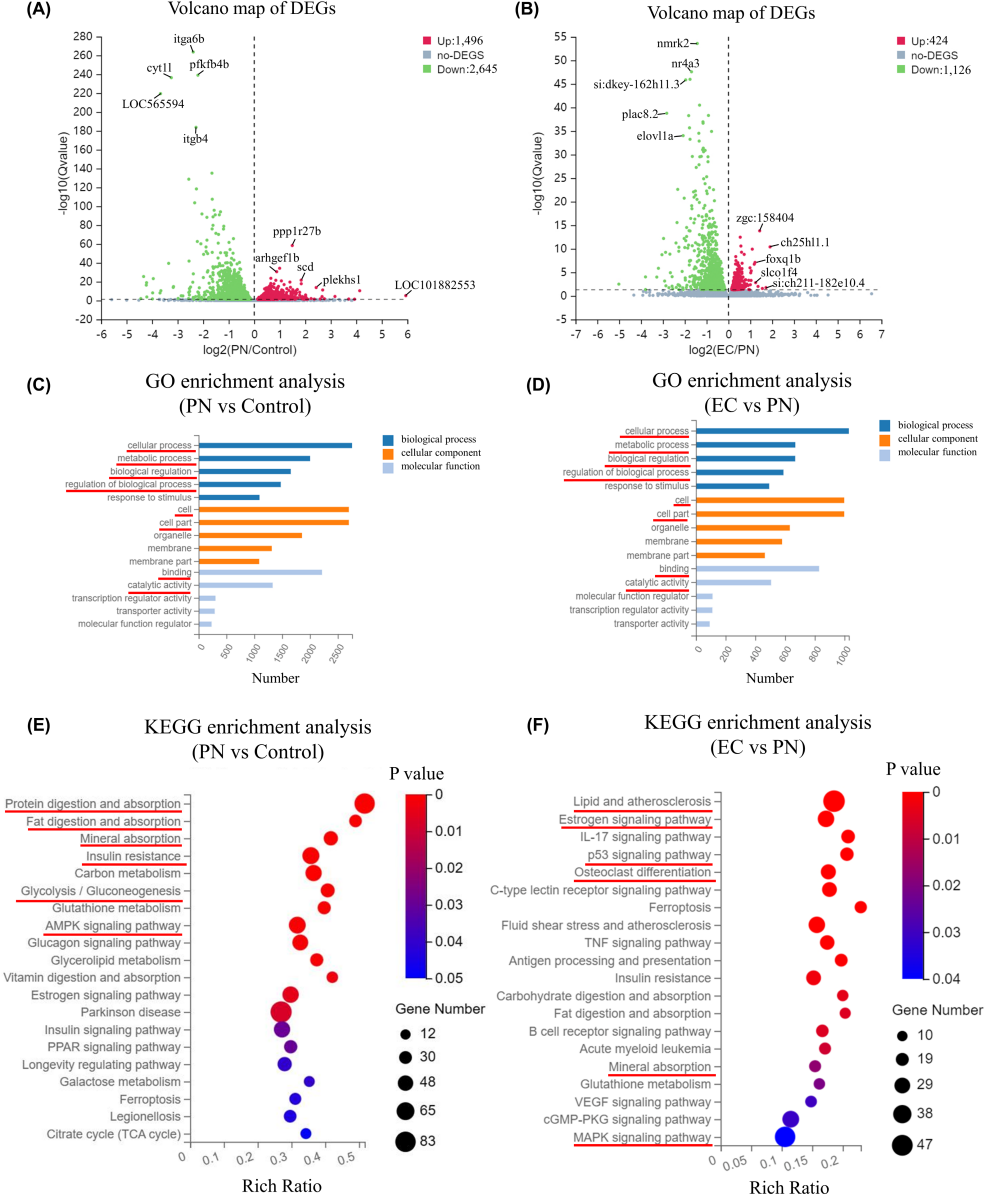
GO and KEGG analyses of DEGs in the 25 μmol/L PN group versus the control group, and the 10 μmol/L EC group versus the 25 μmol/L PN group. (A, B) Volcano map of upregulated and downregulated DEGs. (C, D) GO enrichment analysis of upregulated and downregulated DEGs. (E, F) KEGG enrichment of upregulated and downregulated DEGs. The size of the dots represents the number of genes annotated to the KEGG enrichment, and the colour from red to blue represents a significant degree of enrichment.

The examination of DEGs via RNA sequencing data unveiled substantial changes in gene expression within the control group, the group exposed to 25 μmol/L PN, and the group treated with 10 μmol/L EC. DEGseq algorithms identified 4141 DEGs (1496 upregulated genes and 2645 downregulated genes) between the 25 mol/L PN group and the control group, and 1550 DEGs (424 upregulated genes and 1126 downregulated genes) between the 10 μmol/L EC group and the 25 μmol/L PN group, out of a total of 32,324 genes identified (Figure 6A,B).

### 4.2 Functional annotation of the DEGs

Functional enrichment and GO classification analyses were performed to conduct a more comprehensive analysis of DEGs. The majority of genes associated with cellular components were classified as ‘cell’ and ‘cell part’, according to the GO analysis; conversely, genes associated with molecular functions were primarily involved in ‘binding’ and ‘catalytic activity’. Regarding biological processes, the most represented categories included ‘cellular process’ (2757 or 1029 DEGs), ‘metabolic process’ (2003 or 669 DEGs), ‘biological regulation’ (1656 or 668 DEGs) and ‘regulation of biological processes’ (1476 or 589 DEGs) (Figure 6C,D). The enrichment of DEGs in KEGG pathways was then investigated to further elucidate their potential functions. The KEGG analysis revealed that DEGs in the 25 μmol/L PN group versus the control group were largely enriched in pathways associated with protein digestion and absorption, fat digestion and absorption, mineral absorption, insulin resistance and glycolysis/gluconeogenesis, as well as the AMPK signalling pathway. For DEGs in the 10 μmol/L EC group versus the 25 μmol/L PN group, the most significant enrichment was observed in pathways associated with lipids and atherosclerosis, oestrogen, p53, osteoclast differentiation and mineral absorption, as well as the MAPK signalling pathway (Figure 6E,F).

### 4.3 Identification of DEGs that showed reversed expression on treatment

**FIGURE 7:**
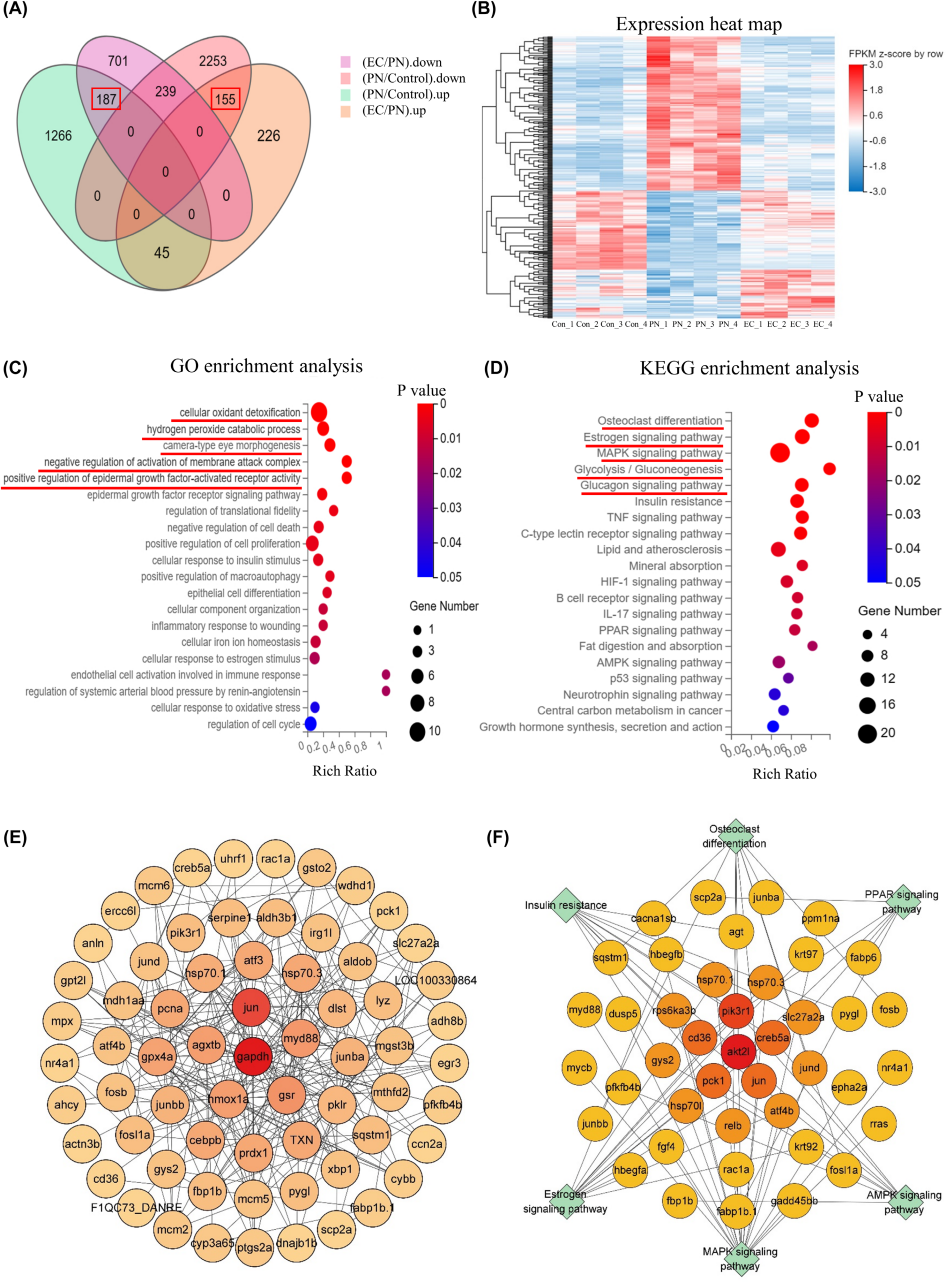
Profile of changes in EC‐induced expression. (A) Venn diagram showing reversal of expression in 342 DEGs. (B) Heat map showing reversed expression of DEGs. Heat map showing changes of expression of the 342 DEGs in the control group, 25 μmol/L PN group and 10 μmol/L EC group. (C) GO enrichment scatterplots of the 342 reversed genes. (D) KEGG enrichment analysis scatterplots of the 342 reversed genes. (E) PPIN of 342 reversed genes. (F) PPIN of DEGs enriched in the signalling pathways related to skeletal development.

A number of DEGs (*n* = 342) that showed reversal of expression between the 10 μmol/L EC group, 25 μmol/L PN group and the control group were identified (Figure 7A). Expression heat maps of DEGs were used to compare gene expression levels between the 10 μmol/L EC, 25 μmol/L PN and control groups (Figure 7B). GO and KEGG enrichment of these 342 DEGs was investigated to better characterize the 10 μmol/L EC exposure–induced regulation of gene expression in zebrafish larvae and to identify the biological significance of the DEGs. The GO enrichment analysis (Figure 7C) showed that the reversed DEGs were enriched in cellular oxidant detoxification, hydrogen peroxide catabolic process, camera‐type eye morphogenesis, negative regulation of activation of membrane attack complex and positive regulation of epidermal growth factor–activated receptor activity. KEGG analysis showed enrichment of the DEGs in osteoclast differentiation, oestrogen, MAPK, glycolysis/gluconeogenesis and glucagon signalling pathways (Figure 7D). Based on the KEGG‐enriched pathways, we concentrated on six pathways that exhibited a strong association with skeletal development. The PPINs (Figure 7E,F) indicate the interrelations between these DEGs.

### 4.4 Verification of transcriptomic sequencing using RT‐qPCR

**FIGURE 8:**
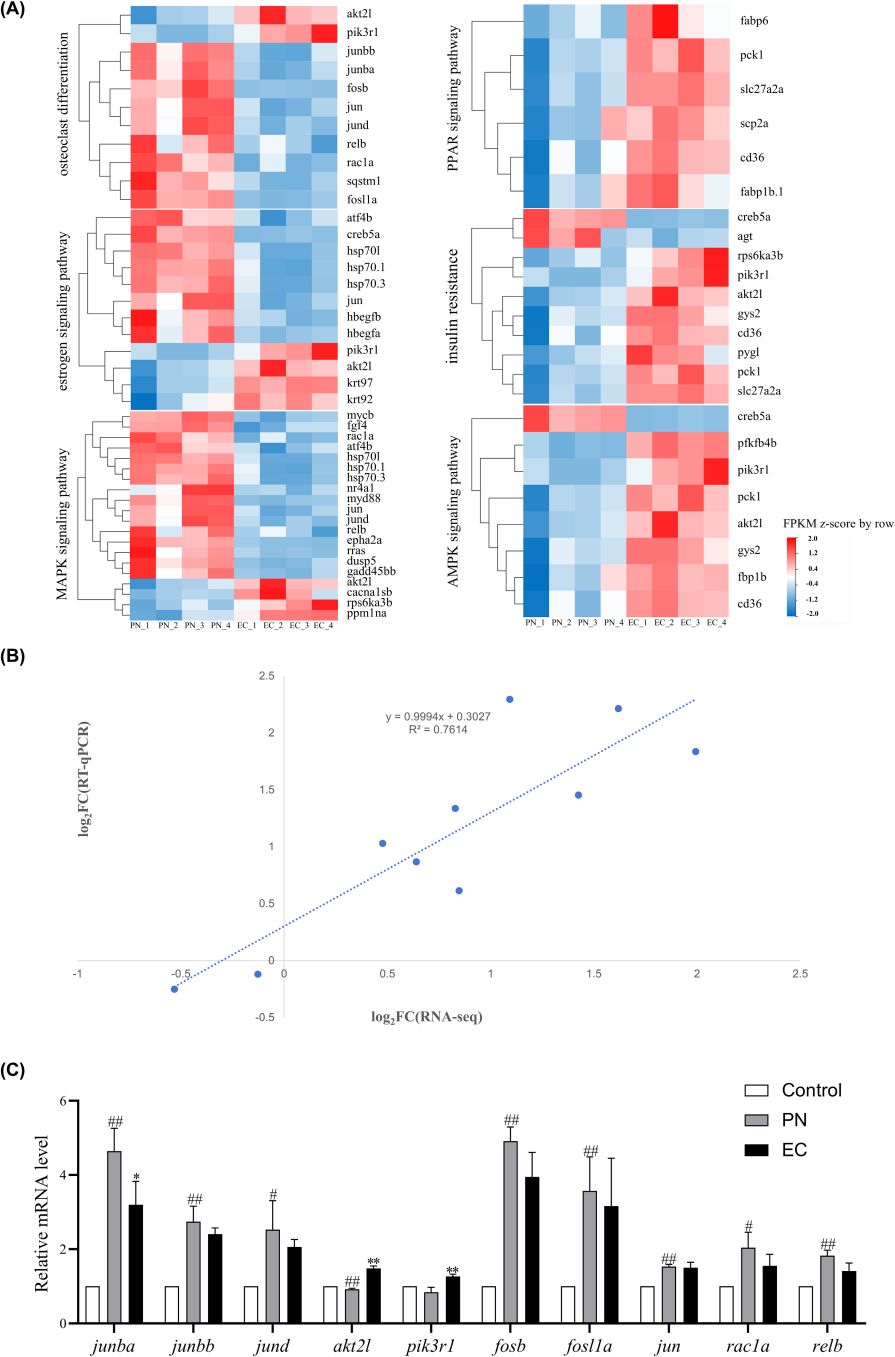
Comprehensive analysis of the key signalling pathways in skeletal development: Expression profiles and transcriptomic verification. (A) Expression heat map of the key signalling pathways related to skeletal development. (B) Verification of transcriptomic data performed by comparing RNA‐Seq results with RT‐qPCR. Dotted lines and dots represent RNA‐Seq and RT‐qPCR data, respectively. *R* ^2^ indicates the correlation strength, with higher values signifying stronger agreement. The *X*‐axis represents the logarithmic (base 2) fold changes in expression levels found using RNA‐Seq. The *Y*‐axis indicates the logarithmic (base 2) value of expression level fold change using RT‐qPCR. (C) Comparison of the expression of 10 genes using transcriptomic analysis and RT‐qPCR (*n* = 3). Compared with the control group, ^#^ *p* < 0.05, ^##^ *p* < 0.01. Compared with the 25 μmol/L PN group, *p < 0.05, **p < 0.01.

To verify the results obtained from the transcriptomic analysis, 10 genes that are characteristic of osteoclasts were examined via RT‐qPCR. While there were slight variations in expression levels, the patterns of expression detected by RT‐qPCR were largely consistent with those identified by high‐throughput sequencing (Figure 8C). The strong correlation between transcriptomic data and RT‐qPCR verification underscores the dependability of RNA‐Seq results. Moreover, a significant positive correlation was noted between the expression fold change of these 10 DEGs from RNA‐Seq and RT‐qPCR results, with a correlation coefficient *R* ^2^ determined to be 0.7614 using linear regression analysis (Figure 8B).

## 5 DISCUSSION

OP is characterized by reductions in both bone mass and density, leading to an increased risk of fracture.^22^ The prevalence of OP is rising because of an ageing population, which presents a serious threat to public health.^23^ PN is commonly used in clinical practice to alleviate inflammation; however, prolonged use may result in secondary OP that adversely affects patient outcomes and well‐being.^24^ To address concerns about model comparability, we employed a zebrafish model treated with 25 μmol/L PN, an established method for inducing osteoporosis in zebrafish larvae supported by multiple studies.^25^, ^26^, ^27^ Our experiments confirmed this concentration as optimal through comparative studies with varying concentrations, where lower doses (e.g. 5 μmol/L) were insufficient to induce the desired osteoporotic changes effectively. Mortality rates of zebrafish larvae under different PN concentrations were systematically monitored, with 25 μmol/L PN inducing significant osteoporosis while maintaining an acceptable mortality rate, ensuring observed phenotypes were due to osteoporotic changes rather than generalized toxicity. Furthermore, ED, a bisphosphonate with a reputation for controlling bone metabolism by encouraging osteoclast death and osteoblast development, is currently a long‐term, minimally invasive method of increasing bone density.^28^, ^29^ In this study, a positive drug group treated with 15 μg/mL ED was used to verify the effectiveness of EC in treating GIOP.

Osteoblasts form the organic bone matrix while osteoclasts degrade bone in a dynamic renewal process that maintains bone health and resilience.^23^ Any disturbance in this equilibrium, particularly when bone breakdown exceeds bone formation, can increase the risk of OP.^30^ Our phenotypic analysis revealed that PN‐exposed zebrafish larvae exhibited a decrease in calcified bone content, aligning with previous research findings.^31^, ^32^ In addition, the KEGG analysis showed that DEGs identified after PN treatment were concentrated in pathways associated with mineral absorption. This suggests that exposure to PN may result in reduced bone density by negatively impacting mineral absorption, reducing bone strength and raising the likelihood of fractures. These findings are in agreement with those reported by Ikeda et al.^33^ Furthermore, we observed that pathways responsible for protein digestion and absorption were significantly associated with the DEGs. This emphasizes that PN can raise the likelihood of fractures by reducing protein synthesis and intensifying protein degradation. This finding supports the hypothesis that OP necessitates higher protein turnover.^34^, ^35^ In addition to protein metabolism, pathways related to lipid metabolism, insulin sensitivity and carbohydrate processing, particularly those involving fat digestion and absorption, insulin resistance and glycolysis/gluconeogenesis, were markedly affected in zebrafish with OP. Variations in the expression of genes within these pathways emphasize their connection to diseases linked to OP, including insulin resistance and type II diabetes mellitus.^36^ Our results indicate that exposure to PN can significantly alter crucial metabolic processes, thereby compromising bone integrity and increasing the risk of fractures. These findings are consistent with our physiological monitoring of zebrafish larvae, which revealed corresponding detrimental effects on bone health and overall physiological function, including reduced locomotion distance and decreased heart rate, blood flow and blood flow velocity.

EC, a compound used in traditional Chinese medicine, is gaining increasing attention for its pivotal contributions in preventing OP, protecting cartilage and reducing inflammation.^37^ When the 10 μmol/L EC group and the 25 μmol/L PN group were compared, it was found that DEGs were primarily involved in pathways associated with lipid metabolism and atherosclerosis. This finding suggests a potential mechanism by which EC might protect against OP in addition to linking high total cholesterol and low‐density lipoprotein cholesterol levels to an increased risk of bone loss and fractures.^38^ A study by Islam et al. demonstrated a significant link between EC and estrogenic activity.^39^ Additionally, a lack of oestrogen is associated with OP, consistent with earlier research.^40^ It has also been reported that a lack of oestrogen increases the production of pro‐inflammatory factors, thereby facilitating osteoclast differentiation and accelerating the advancement of OP.^41^ Similarly, the KEGG analysis in the present study revealed significant enrichment of the DEGs in osteoclast differentiation and oestrogen signalling pathways, indicating that the anti‐GIOP action of EC was at least partially mediated by raising oestrogen levels and preventing osteoclast development. However, further research is required to fully support this hypothesis.

We focused our analysis on genes that showed reversal in expression in several signalling pathways, including pathways associated with osteoclast differentiation, oestrogen and insulin resistance, as well as the MAPK, PPAR and AMPK signalling pathways, that are important for bone development and associated with OP, as reported in recent studies and the KEGG reference pathways. Guo et al. have underscored the crucial function of osteoclast differentiation pathway in skeletal development, revealing the molecular mechanisms that could be targeted to modulate this process and thereby preserve bone integrity.^42^ Equally important to bone development is the oestrogen signalling pathway. Cheng et al. have underscored the role of oestrogen in regulating bone density, highlighting how its deficiency could contribute to postmenopausal OP, and suggesting the potential that oestrogen replacement therapy holds in preventing and mitigating bone loss.^43^ The MAPK signalling pathway plays a critical role in bone cell dynamics, as revealed by Ma et al. This includes direct effects on osteoclast and osteoblast functions, which are essential for bone growth and maintenance, especially in situations where oestrogen levels are low.^44^ Furthermore, recent research by Wong et al. clarified the function of insulin resistance in bone health by showing how it affects osteoblast development, which is compromised, and increased bone resorption, which lowers the quality of bone. These results highlight the complex connections between skeletal strength and metabolic disorders.^45^ Li et al. have reported the importance of the PPAR signalling pathway in bone development, demonstrating its influence on the fate of mesenchymal stem cells and the potential of this pathway as a therapeutic target in enhancing bone density.^46^ Guo et al. studied the AMPK signalling pathway and have reported its role in bone remodelling, emphasizing its capacity to suppress osteoclast activity and thus promote skeletal health.^47^

Our PPIN analysis of these six pathways in our study revealed that *fosb* ^48^ and *fosl1a*,^49^ integral components of the AP‐1 complex, are crucial for osteoclast differentiation,^50^ and that genes characteristic of osteoclasts were associated with the NF‐κB,^51^ JNK/c‐Jun^52^ and PI3K/Akt^53^ signalling pathways, all of which contribute to osteoclast differentiation. Significant reductions in the expression of osteoblast‐characteristic genes and increases in the expression of osteoclast‐characteristic genes were observed, both of which are consistent with the phenotype generated by PN.^54^, ^55^ By downregulating the genes associated with osteoclastic phenotype, EC may be able to cure GIOP by reducing the differentiation of osteoclasts.^43^ Interestingly, our KEGG pathway analysis revealed distinct yet complementary actions of PN and EC. PN primarily affects pathways related to protein digestion and absorption, fat digestion and absorption, mineral absorption, insulin resistance, glycolysis/gluconeogenesis and the AMPK signalling pathway. In contrast, EC modulates pathways involved in lipids and atherosclerosis, oestrogen signalling, p53 signalling, osteoclast differentiation, mineral absorption and the MAPK signalling pathway. These findings suggest that EC does not directly counteract PN's glucocorticoid activity but provides protection through specific molecular mechanisms. Huang et al. showed that EC mitigates glucocorticoid‐induced suppression of osteogenic differentiation via the PI3K/AKT/RUNX2 signalling pathway, suggesting EC alleviates the inhibitory effects on bone formation without directly opposing glucocorticoids.^32^ Moreover, a study on EC and icariin in a zebrafish osteoporosis model demonstrated that EC promoted cranial mineralization and effectively prevented PN‐induced osteoporosis without antagonistic effects.^15^ Collectively, our data and supporting studies indicate that EC protects against PN‐induced osteoporosis by modulating bone remodelling pathways, crucial for clinical applications. This implies that co‐treatment with EC can mitigate the adverse skeletal effects of glucocorticoids without compromising their therapeutic efficacy.

## 6 CONCLUSIONS

An experimental model was created in zebrafish larvae using the GIOP method. The doses and duration of prednisolone administration were adjusted to optimize the model. The effectiveness of EC in reducing bone loss was then evaluated. Quantitative analysis using alizarin red S staining, calcein immersion, fluorescence imaging and statistical methods revealed the skeletal development of zebrafish larvae and verified the efficacy of EC. EC could significantly inhibit bone loss by modulating osteoclast activity. Specifically, EC affected several critical pathways involved in bone metabolism, such as pathways associated with osteoclast differentiation, oestrogen and insulin resistance, together with the MAPK, PPAR and AMPK signalling pathways. The results demonstrate that EC can protect against GIOP‐induced osteoporosis. Hence, additional exploration of EC for OP treatments is necessary, with a focus on understanding its mechanisms and prospective applications in more complex models. Ultimately, the possibility of conducting human clinical trials should also be taken into consideration.

## AUTHOR CONTRIBUTIONS

**Xiaoyang Zhou:** Data curation (equal); formal analysis (equal); investigation (equal); methodology (equal); validation (equal); writing – original draft (equal); writing – review and editing (equal). **Kai Lian:** Conceptualization (equal); funding acquisition (equal); writing – review and editing (equal). **Junjie Jia:** Data curation (equal); investigation (equal); supervision (equal). **Xue Zhao:** Investigation (equal); methodology (equal); supervision (equal). **Peng Duan:** Conceptualization (equal); methodology (equal); supervision (equal); writing – original draft (equal); writing – review and editing (equal). **Jiaolong Huang:** Conceptualization (equal); data curation (equal); methodology (equal); writing – review and editing (equal). **Yihua Shi:** Data curation (equal); methodology (equal); supervision (equal).

## FUNDING INFORMATION

This work, in part, was supported by grants from the Hubei Province Key Research and Development Program (No. 2022CFD010, No. 2022 BCE021), the Foundation of Health Commission of Hubei Province (No. WJ2023M162) and the Scientific and Technological Project of Xiangyang City of Hubei Province (No. 2022YL24A).

## CONFLICT OF INTEREST STATEMENT

The authors declare no competing interest.

## Data Availability

The data that support the findings of this study are available from the corresponding author upon reasonable request.
